# Dietary Intake, Biological Status, and Barriers towards Omega-3 Intake in Elite Level (Tier 4), Female Athletes: Pilot Study

**DOI:** 10.3390/nu15132821

**Published:** 2023-06-21

**Authors:** Matthew P. Hooks, Sharon M. Madigan, Jayne V. Woodside, Anne P. Nugent

**Affiliations:** 1 School of Biological Sciences, Institute for Global Food Security, Queen’s University Belfast, Belfast BT9 5DL, Northern Ireland, UK; anne.nugent@ucd.ie; 2 Sport Ireland Institute of Sport, D15 Y52H Dublin, Ireland; smadigan@instituteofsport.ie; 3 Sport and Human Performance Research Centre, University of Limerick, V94 T9PX Limerick, Ireland; 4 Department of Physical Education and Sport Sciences, University of Limerick, V94 T9PX Limerick, Ireland; 5 Centre for Public Health, Institute for Global Food Security, School of Medicine, Dentistry and Biomedical Sciences, Queen’s University Belfast, Belfast BT9 7BL, Northern Ireland, UK; j.woodside@qub.ac.uk; 6 UCD Institute of Food and Health, School of Agriculture and Food Science, University College Dublin, D04 V1W8 Dublin, Ireland

**Keywords:** omega-3, dietary intake, omega-3 index, fatty acids, female athletes, barriers

## Abstract

Omega-3 polyunsaturated fatty acids (*n*-3 PUFA) have unique properties which benefit athlete populations. The literature investigating NCAA collegiate, rugby sevens and German endurance athletes indicates suboptimal *n*-3 PUFA dietary intake and biological status. The aims of this study were: (i) to explore the dietary intakes and FA profiles of elite level, team-based, female athletes and (ii) to understand perceived barriers towards achieving *n*-3 dietary guidelines. A total of 35 athletes (24.8 ± 4.5 years) completed both a questionnaire and a finger prick test. All the participants reported consuming fish and seafood over the previous six months however only nine athletes consumed ≥ 2 servings of fish per week. Four participants reported using an *n*-3 supplement. The mean omega-3 index (O3I; including supplementers) was below target levels of >8% (5.19 ± 0.86%). O3I was significantly higher (*p* < 0.001) in those consuming ≥ 2 servings of fish per week and/or supplements (5.91 ± 0.81%) compared with those who did not (4.82 ± 0.63%). The main barriers reported by those not consuming two servings of fish per week were sensory (n = 11; 42%), cooking skills (n = 10; 38%) and knowledge of *n*-3 benefits (n = 7; 27%). The current study shows that elite level female athletes present with suboptimal *n*-3 dietary intake and O3I due to their food preferences, cooking skills and *n*-3 knowledge.

## 1. Introduction

The ever-growing professionalism of female sport along with increasingly congested fixtures lists has led to the increased focus of players and support staff on female specific nutritional strategies to maximise both performance and recovery [1,2]. The sex differences regarding recovery from an exercise stimulus have previously been highlighted in the literature, with some evidence showing women taking longer to return to a pre-exercise state, i.e., cardiac and respiratory recovery and prolonged muscle soreness [3,4,5]. The physiological sex differences along with the rapid growth of female sport have pushed many athletes to explore specific nutritional strategies that will aid the recovery process and, therefore, reduce the risk of injury and underperformance throughout the season [6,7]. This attempt to maximise recovery has subsequently driven research into more novel nutritional strategies, such as the application of omega-3 polyunsaturated fatty acids (*n*-3 PUFA) within a sporting context [8].

*n*-3 PUFAs have unique structural and signalling properties within the body. Specifically, it is their anti-inflammatory properties that have spiked interest [9]. *n*-3 PUFA are characterised by a double bond at the third carbon from the methyl end of the carbon chain. The three most well-known *n*-3 PUFA are alpha-linoleic acid (ALA; 18:3*n*-3), eicosapentaenoic acid (EPA; 20:5*n*-3) and docosahexaenoic acid (DHA; 22:6*n*-3). ALA is a plant derived, parent fatty acid (FA) which acts as a precursor for the marine-derived EPA and DHA [10]. The process of EPA and DHA synthesis from ALA requires a series of elongase, desaturase and β–oxidation reactions [11]. However, this process of endogenously synthesising EPA and DHA is very limited in humans, with <1% and <9% of ALA being converted to DHA in males and females, respectively [12,13]. EPA and DHA can, therefore, be considered as essential and should be sourced in the diet from food and/or supplements. The European Food Safety Agency (EFSA) recommends a daily intake between 250 mg and 500 mg of EPA and DHA combined, which equates to around two servings of oily fish per week [14].

The *n*-3 status of an individual can be gauged through the assessment of *n*-3 dietary intake and the biological analysis of the blood FA profile, which is represented by the omega-3 index (O3I). The O3I is a measure of the EPA and DHA as percentage of total FAs in the erythrocyte membranes [15]. A systematic review carried out in 2016 investigating the *n*-3 status globally revealed considerable variation in circulating EPA and DHA status, with low O3I (≤4%) identified in multiple locations around the world, including the UK and Ireland [16]. The endpoints used to categorise different levels of O3I are benchmarked to the coronary heart disease (CHD) literature. Harris et al. reported that people who are at highest risk of CHD had an O3I of <4%, while those with a moderate risk had O3I values of 4–8%, and the values for the lowest risk group were ≥8% [17]. When combining the current *n*-3 observational studies with *n*-3 dose response intervention studies, both males and female athletes (NCAA, professional rugby, winter and summer Olympic sports) present with suboptimal omega-3 index (<8%) [18,19,20,21].

There are numerous factors which contribute to the interindividual variability in the omega-3 index, including sex, age, body weight and training status [22,23,24]. The previous observational research investigating *n*-3 dietary intake and FA blood profiles has indicated that elite level athletes are not reaching dietary intake guidelines and present with suboptimal O3I. However, the literature investigating exclusively on elite/ international level female athletes is very limited with small sample sizes and has failed to collect data on the barriers towards reaching *n*-3 guidelines [18]. Therefore, the aim of this study was to (i) further explore the dietary intake and FA profile of elite international level (tier 4), team-based, female athletes and increase our understanding around (ii) perceived barriers towards reaching *n*-3 dietary guidelines within this population.

## 2. Materials and Methods

### 2.1. Participants

The participants were recruited from the Sport Ireland Institute (SII). SII is a government funded organisation that provides sports science, medical and performance support to Irish athletes aiming to qualify and medal at major competitions such as the Olympic, Paralympic, and other games. The target subject pool was elite international level female athletes participating in team-based sports (hockey and cricket), where the competition format (i.e., Olympics and world cup) of the sport is characterised by short recovery windows between matches (<72 h). The priority for athletes competing in these competitions is to minimise exercise-induced muscle damage (EIMD) and speed up the recovery process in an attempt to reduce the risk of injury and underperformance [6,7]. The key eligibility criteria were that recruits were female and must be participating at a tier 4 level as defined by the published participant classification framework, i.e., a national team competing in international leagues/tournaments, placed within the top 4–300 in the world, and highly proficient in the required skills [25]. All cricket and hockey participant data were collected in the morning at the start of their pre-season introductory camp based at SII in December and February, respectively. Ethical approval was granted by The Faculty of Medicine, Health and Life Sciences Research Ethics Committee, Queens University Belfast (MHLS 23_25). Informed consent was obtained from all participants prior to commencing the study.

### 2.2. Study Design

This was a cross-sectional, observational study. The participants were asked to complete an online *n*-3 questionnaire and provide a dried whole blood sample for analysis.

### 2.3. Omega-3 Questionnaire

The short online questionnaire (Supplemental File S1) was adapted from a previously validated 21-item *n*-3 PUFA food frequency questionnaire (FFQ) [26]. The 21 core questions assessed *n*-3 dietary intake (quantity and frequency) over the previous six months with an additional three questions focusing on barriers towards *n*-3 PUFA intake, reasons for supplementing and source of *n*-3 information. The FFQ which formed the base of the current questionnaire is a validated tool used to assess *n*-3 dietary intakes and has previously been used in published research of a similar nature [18,20,26].

The core questionnaire assessed the participants’ *n*-3 PUFA dietary intake over the previous six months by collecting consumption frequencies of a range of *n*-3 PUFA-rich foods, specifically seafood, walnuts, rapeseed oil, flaxseed, flaxseed oil, chia seeds and vegetable margarine, and information on *n*-3 supplements from those who indicated they were users, including the type, brand, dose, and frequency. The list of different fish and seafood was adapted according to local fish consumption patterns [27,28]. The *n*-3 PUFA intakes, specifically ALA, EPA and DHA, were subsequently calculated based on the types of foods which were consumed by participants, the reported quantity of food, the frequency of consumption and the average FA content of the reported foods. The FA content of foods was determined using published food composition tables [29]. If a participant specified that they consumed a variety of different species of fish, the reported quantity and frequency was divided by the number of species reported. This method for calculating *n*-3 PUFA intake has been used previously to validate the 21-item FFQ [26].

The additional question used to assess perceived barriers towards *n*-3 intake were based on previous findings from a systematic review which investigated the characteristics of seafood consumers as well as the influences on seafood consumption in Europe, USA, Canada, Australia, and New Zealand [30].

### 2.4. Omega-3 Biological Status

The second part of the study required the participants to provide a dried blood spot (DBS). The only condition was that they had to refrain from *n*-3 consumption 12-h before providing a sample. The whole blood sample was collected from each participant’s index finger onto a OmegaQuant collection card by an accredited performance nutritionist or team doctor. The designated spot on the collection card was pre-treated by OmegaQuant with a proprietary antioxidant treatment called OxyStop which prevents oxidative loss of PUFAs. The sample was allowed to dry for 15 min before being sent the same day to a commercial laboratory (OmegaQuant, University of Stirling, GB) for analysis. The laboratory transfers one punch from the DBS card (10 uL of blood) into a vial which undergoes a series of steps including heating, cooling, vortexing and centrifuging leading to the separation of layers. The upper layer is then transferred to a sperate vial and undergoes gas chromatography. Further details regarding DBS sampling are outlined in the methodology of a previous study [31]. The individual FA values are calculated on a whole blood basis, whereas the O3I values are calculated for red blood cells specifically.

### 2.5. Statistical Analysis

Data analysis was completed using IBM Statistical Package for the Social Sciences (SPSS) version 28. Descriptive statistics for the continuous data are presented as mean ± standard deviation, and the categorical data are presented as frequencies and percentages. A test for normality was carried out using Shapiro–Wilk’s test. Between group comparisons were completed using an independent samples *t*-test and a one-way, between-groups ANOVA followed by Tukey’s HSD post hoc test. Spearman’s rho non-parametric test was used to assess the relationship between diet and blood variables. A significant difference was detected when *p* < 0.05.

## 3. Results

### 3.1. Participants

A total of 40 elite level, team-based, female athletes were invited to participate in this study, five participants failed to complete both components of the study and were excluded. A total of 35 female athletes (hockey = 20; cricket = 15) with a mean age of 24.8 ± 4.5 years completed both parts of the study and were included in the following analysis.

### 3.2. Diet

**Figure 1:**
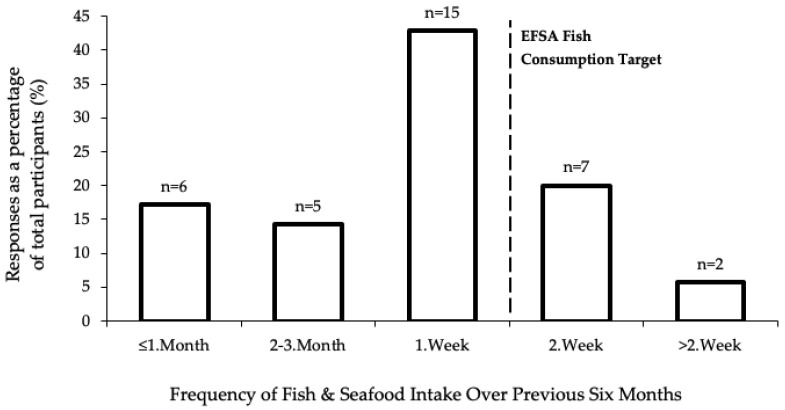
Frequency of fish and seafood consumed over the previous six months; The European Foods Safety Agency (EFSA).

**Figure 2:**
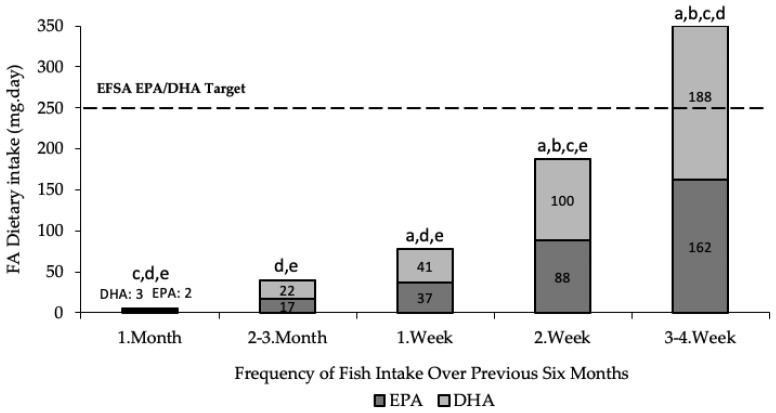
Estimated daily dietary fatty acid intake (excl. contribution from supplements) according to the frequency of fish and seafood consumed over the previous six months. All data presented as means and analysed using one-way between-groups ANOVA followed by Tukey’s HSD post hoc test (*p* < 0.05). a indicates statistically significant difference in frequency of fish intake of versus <1 month; b, versus 2–3 month; c, versus 1 week; d, versus 2 week; e, versus 3–4 week.

All 35 elite level, female athletes reported consuming fish and seafood over the previous six months. Only nine (26%) participants consumed ≥ 2 servings of fish per week (Figure 1). Furthermore, only eight (23%) participants achieved the EFSA guidelines for a combined daily intake of EPA and DHA ≥ 250 mg, and of these eight participants, four reported using an *n*-3 supplement [14]. The EPA and DHA dietary intake (excl. contributions from supplements) is shown in Figure 2. The most common barriers reported by the 26 (74%) participants who reported not consuming ≥ 2 servings of fish per week were sensory (n = 11; 42%), cooking skills (n = 10; 38%), and knowledge of *n*-3 PUFA health and performance benefits (n = 7; 27%). Cod (n = 28; 80%) and salmon (n = 26; 74%) were the most frequently consumed fish and seafood, whereas none of the participants reported consuming herring or mackerel.

The reported sources of ALA consumed by participants included chia seeds (n = 24), walnuts (n = 22), rapeseed oil (n = 20), flaxseed (n = 15) and flaxseed oil (n = 2). The mean daily intake of ALA (810 ± 1280 mg) was below the recommended daily guideline for females (1.1 g per day) as stated by the National Institutes of Health [32]. A total of seven (20%) participants had a daily intake of ALA above the National Institutes of Health guideline [32].

Supplement use was reported by four (11%) participants, with the brand being used for each participant. Three of these participants supplemented daily over the previous six months, whereas the other athlete reported a frequency of 3–4 times per week. Three of the supplementers were consuming < 2 servings of fish per week. The reported reasons for using a supplement were to enhance recovery and for cognitive benefits.

### 3.3. Blood

The mean O3I was 5.19% and ranged from 3.29% to 7.39% (incl. supplementers). Only one participant fell into the high-risk of CHD category based on O31 (<4%). However, none of the participants achieved an O3I of ≥8% (low risk CHD category) [15,16]. The dietary intakes of ALA were not correlated with blood values; however, there was a strong, positive correlation between the calculated dietary intake of EPA and DHA and the measured blood values of EPA, DHA and O3I (Table 1).

**Figure 3:**
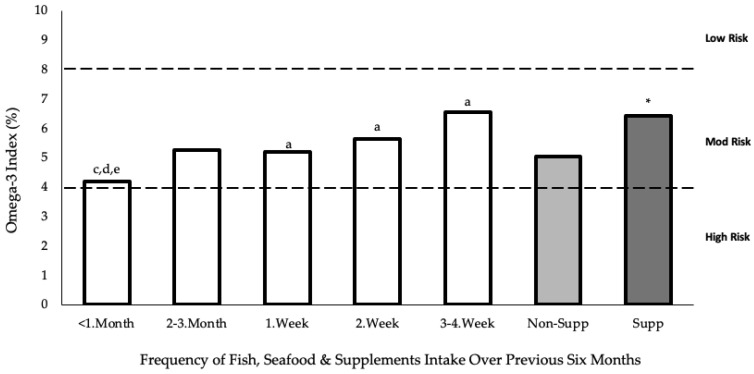
Frequency of fish, seafood and supplement intake against O3I. The O3I data include those who supplemented within that category for fish and seafood intake. Low, moderate, and high ranges are based on the CHD risk factor. Fish frequency versus O3I are presented as means and analysed using one-way between-groups ANOVA followed by Tukey’s HSD post hoc test: a, *p* < 0.05 versus frequency of fish intake of <1 month; c, *p* < 0.05 versus frequency of fish intake of 1 week; d, *p* < 0.05 versus frequency of fish intake of 2 week; e, *p* < 0.05 versus frequency of fish intake of 3–4 week. Supplement status versus O3I was analysed using independent *t*-test: *, *p* < 0.05 versus non-supplementers.

The participants who reported consuming ≥ 2 servings of fish and seafood per week and/or taking supplements had a significantly (*p* < 0.001) greater O3I than those who consumed < 2 servings of fish and seafood per week and/or did not consume a supplement (5.91 ± 0.81% and 4.82 ± 0.63% respectively). The mean O3I values were significantly (*p* < 0.001) greater in those who supplemented (n = 4) compared with non-supplementers (n = 31), with levels of 6.42 ± 0.71% and 5.04 ± 0.75%, respectively. Considering the relationship between O3I and frequency of fish intake, Figure 3 shows a statistically significant (*p* = 0.001) difference between the groups. Post hoc comparisons using Tukey’s HSD test indicate that the mean O3I of those who consumed fish and seafood < 1 month (4.18 ± 0.48%) was significantly lower than the O3I of those who consumed fish and seafood 1 week (5.19 ± 0.64%, *p* = 0.035), 2 week (5.64 ± 0.88%, *p* = 0.005) and 3–4 week (6.55 ± 0.98%, *p* = 0.002). There were no other significant differences detected, including between <1 month and 2–3 month (5.25 ± 0.60%).

## 4. Discussion

This study investigated the recent *n*-3 dietary intake, biological status, and barriers against reaching current *n*-3 guidelines within elite international level (tier 4), team-based, female athletes. The results show that this population does not meet the *n*-3 PUFA dietary guidelines, which is reflected in the athletes’ suboptimal O3I. The main reasons reported for athletes not achieving the *n*-3 guidelines were sensory barriers (taste and smell) towards fish, inadequate cooking skills and knowledge of *n*-3 benefits. To our knowledge, this is the first study to combine both *n*-3 dietary and blood FA analyses with perceived barriers in elite international level female athletes.

### 4.1. Diet

Previous research conducted on a global scale identified that <20% of males and females achieve the recommended daily intake benchmark of ≥250 mg of EPA and DHA combined [14,16]. To achieve these guidelines, it is suggested that individuals require two servings of fish per week [33]. In the current study, 23% of participants reported consuming ≥ 2 servings of fish and seafood per week, which is lower than the levels reported by previous research in athlete populations, specifically, NCAA collegiate athletes (39%) [20]. Although the current study found a lower percentage of participants meeting dietary intake guidelines, the mean daily intake of EPA and DHA combined was double that previously measured in female collegiate level athletes (250 mg and 124 mg, respectively) [23]. A possible reason for this contradiction within the findings could be the small sample size of the current study, the different combinations of fish consumed, inter-individual variability in the FA composition of fish and the possible impact of training status (i.e., elite vs collegiate) [24,34]. Furthermore, the current intake of elite level female athletes is also higher than previous values collected for the general Irish population aged 18–35 years (201.8 mg) [27]. Although the participants reported higher *n*-3 PUFA intakes, they are still at the lower limit of the EFSA guidelines for EPA and DHA (EFSA upper limit: 5 g·day) [14]. The collective *n*-3 PUFA literature provides evidence for promising effects on cognitive functions, physical performance, management of exercise-induced muscle damage and general health [35]. Specifically, within females it has been linked to a reduction in the severity of premenstrual syndrome symptoms [36]. Therefore, it should be a priority for athletes and support staff to further increase EPA and DHA intake [37,38,39,40,41].

The species of fish consumed is an important consideration in terms of *n*-3 PUFA dietary intake, with the fatty/oily fish providing a greater source of *n*-3 [14]. The most frequently consumed fish were cod and salmon, whereas there was little to no reported intake for other key sources of *n*-3 in addition to salmon, such as trout, herring, and mackerel. There were nine (26%) participants who reported consuming no dietary (incl. supplements) source of EPA and DHA. These results are similar to previous findings for collegiate level athletes [20]. It is, therefore, suggested that athlete support personnel should first educate athletes regarding the types of fish which are high in EPA and DHA and also consider promoting the inclusion of other quality *n*-3 food sources which can improve the *n*-3 index, such as enriched foods and supplements [19,42,43].

The mean O3I for supplementers (6.55 ± 0.98%) was significantly higher than that of non-supplementers (5.04 ± 0.75%). The importance influence of supplementation on O3I and the consequent CHD risk factor has previously been shown in the literature, with fewer supplementing female rugby players presenting in the high-risk category compared with their non-supplementing counterparts (39.1% and 45.5%, respectively) [18]. Moreover, only 11% of the current cohort reported that they had consumed an *n*-3 supplement over the previous six months, which is a much lower proportion than previously reported for elite international level, team-based female athletes (73%) [18].

### 4.2. Blood FA Profile

The O3I of the elite level female athletes under study here (5.19 ± 0.86%) was suboptimal according to the CHD literature and below the levels found in general population data collected in Europe (6.96 ± 2.15%) [15,17,40]. However, the O3I values from this study are higher than previous values collected for Canadian, elite international level, team-based female athletes (n = 15; 4.61 ± 2.40%) [18]. Furthermore, the FA data from the current study show that no participant reached the O3I target of ≥8%, including those who consumed two or more portions of fish per week and/or consumed *n*-3 PUFA supplements over the previous six months. This is reflective of the baseline O3I from a dose response study within Spanish male and female elite level athletes [19]. These data are consistent with the conclusions of McDonnell et al., who suggest that the current public health advice of two servings of fatty fish per week, or one serving daily of a *n*-3 supplement will not produce an O3I of ≥8% [44]. Evidence from Japanese dietary intakes, where the average O3I is ~8%, suggests that three or more servings per week of fish are required to achieve these levels [45]. Moreover, the EPA + DHA content of farmed Atlantic salmon has declined by >60% since 2005, thus providing a possible explanation for the current inadequate and outdated guidelines [46]. Other research has also shown significant inter-individual variation in the FA composition of fish and has suggested it may be recommended to top-up dietary intake with a quality daily supplement which is high in EPA + DHA (>500 mg) [34,47]. This guidance is supported by our findings that the participant with the highest O3I (7.39%) was the only individual to also report that they consume two servings of fish and supplemented 3–4 times per week.

### 4.3. Barriers

The main barriers reported by those who consumed < 2 portions of fish per week were sensory barriers (n = 11; 42%), practical cooking skills (n = 10; 38%) and knowledge of *n*-3 benefits (n = 7; 27%). This is an important finding because the recommendation from previous literature which identified suboptimal dietary intake was for support personnel to promote fish and seafood consumption to increase *n*-3 status [20]. However, the reported barriers in this study, specifically the taste and smell of fish, suggest that this general advice isn’t appropriate for everyone and alternative nutritional interventions aimed at improving O3I should be adopted, such as the inclusion of *n*-3 enriched foods and/or good quality batch-tested supplements. Both supplements and *n*-3 enriched foods such as eggs, poultry and drinks have been shown to be effective ways to promote the *n*-3 status of an individual [42,48,49]. Moreover, cooking skills were also highlighted as a major barrier. Upskilling athletes in the kitchen might also be an alternative approach to addressing suboptimal dietary intake and *n*-3 status. Cooking workshops not only develop the basic food preparation skills of the athlete but also provide an invaluable opportunity to educate, thus giving the athlete the skills and knowledge to execute the desired strategy. Furthermore, this strategy should be introduced with younger athletes as developing these skills early in their career will improve their ability to meet their nutritional requirements [50]. Moreover, this strategy is especially important in Olympic sports, which might not be as well-resourced as professional rugby and football teams, and therefore, follow a more hybrid approach towards food provision, with athletes being responsible for preparing some meals [51]. The third barrier reported was the lack of knowledge regarding *n*-3 and that the participants were not aware of the benefits which *n*-3 can elicit. Together, the identification of these barriers provides the athlete support personnel with the information to help design successful nutrition interventions targeting suboptimal *n*-3 intake i.e., incorporation of various *n*-3 sources, development of cooking skills and educating the athlete.

Few participants in the current study reported consuming *n*-3 supplements, even though they are shown in the previous literature to successfully increase O3I, and therefore, represent a suitable alternative for those who do not include an adequate amount of fish in their diet [52,53,54]. The current study did not collect information on why athletes were not using supplementation. However, previous *n*-3 studies have reported a perception within the adult population that food alone can be sufficient, and supplementation is not required [43]. Furthermore, supplementation within elite level athletes is not straightforward due to the anti-doping risk associated with supplements, specifically the fear that the supplement contents are different from those stated on the label [55]. The variation in *n*-3 supplement manufacturing standards is highlighted by a previous study which analysed 45 *n*-3 supplements and found that the EPA and DHA content was less than the stated dose in 70% of products [56]. Furthermore, high levels of oxidised lipids, a sign of lipid degradation, were found in more than 80% of 35 *n*-3 supplements, and only 8% met international peroxide and total oxidation standards [57]. To maximise safety and ensure supplement standards are met, elite level athletes should choose a third-party batch-tested supplement from a reputable brand and consider recognised supplementation protocols (e.g., dose, duration, baseline O3I, training status, chemical form, and *n*-3 PUFA composition) as described elsewhere [49,58,59].

### 4.4. Strengths and Limitations

A major benefit of the current study was that both the finger prick test and FFQ allowed samples to be collected quickly and easily in the field with minimal distress for participants. This is crucial when working in elite level environments. The methods of this study are closely aligned with those of previous studies assessing *n*-3 dietary intake and biological status, which strengthens any comparisons made between findings [18,20]. Moreover, the current study has reproduced the findings in the literature, which shows the reliability and validity of the inexpensive FFQ through positive correlations between blood FA data and measured *n*-3 PUFA intake [20,26,60]. Previous real-world testing using the finger prick test found high levels of accuracy, with predicted O3I falling within ±1% of measured levels [31].

The limitations of this study include the small sample size; however, participant homogeneity was maintained (i.e., elite level, female athletes from team sports) so that stronger conclusions could be drawn from results. Furthermore, the participant classification framework clearly states that research within tier 4 athletes is challenging due to the limited numbers, with only ~0.003% to 0.006% of the global population falling into this classification [25]. However, quality research within this population can still provide valuable conclusions with high ecological validity [25].

The current study included participants only from team sports. Therefore, it is hard to generalize our conclusions to athletes in other types of sport (i.e., individual, endurance, track & field etc.) who might have different cultures and socio-economic backgrounds and, therefore, different dietary habits [61]. Future research should involve a range of tier 4/5 female athletes from a variety of sports which would allow comparisons between sports and provide a greater understanding of the greater sporting landscape. Moreover, as cooking skills were identified as a major barrier to adherence to nutrition guidelines, future research may look to develop an intervention which targets this and measures changes to dietary intake as a primary outcome. The current study did not collect information on why participants were not supplementing. Therefore, we could only speculate that the perception of supplementation not being necessary and anti-doping risk were contributing factors. However, earlier research that identified a decreasing trend in supplement use among Olympic athletes also referred to this anti-doping risk [62].

## 5. Conclusions

The current study investigated the dietary intake, biological status, and barriers towards *n*-3 PUFAs and discovered that elite level, team-based, female athletes do not meet the current dietary guidelines of two servings of fatty fish per week and/or 250–500 mg daily of EPA and DHA combined. No athletes reached the current O3I target of >8% (lowest risk of CHD), and the main barriers to reaching *n*-3 dietary intake guidelines reported by athletes were sensory (taste and smell), cooking skills and knowledge of *n*-3 health and performance benefits. Overall, this study provides athlete support personnel working within this current classification of athletes the information to design successful nutritional interventions which focus on performance and health through *n*-3 intake.

## Figures and Tables

**Table 1:** *n*-3 PUFA composition of the dried blood spots and dietary questionnaire (incl. supplementers), presented as mean ± SD.

		Blood Sample (% *w*/*w*)	Total Intake (g·day)	Spearman’s Rho
ALA		0.44 ±0.15	0.81 ± 1.28	r = 0.31
EPA **		0.66 ± 0.25	0.15 ± 0.29	r = 0.60
DHA **		2.73 ± 0.62	0.10 ± 0.15	r = 0.59
O3I	EPA + DHA **	5.19 ± 0.86	0.25 ± 0.44	r = 0.76

Spearman’s rho non-parametric test was used to assess the relationship between diet and blood variables (*p* < 0.05). ** denotes sig. correlation between FA intake and red blood cell concentrations at *p* < 0.001.

## Data Availability

Research data for the current study has not been made public.

## Supplementary Materials

The following supporting information can be downloaded at: https://www.mdpi.com/article/10.3390/nu15132821/s1, File S1: Omega-3 Fatty Acids Questionnaire.
