# Supplementary material for: Dietary Intake, Biological Status, and Barriers towards Omega-3 Intake in Elite Level (Tier 4), Female Athletes: Pilot Study
Source: Nutrients. 2023 Jun 21;15(13):2821. doi: 10.3390/nu15132821 (PMC10343224; doi:10.3390/nu15132821)
Supplement: Supplementary file 1 [file nutrients-15-02821-s001.zip › nutrients-2418261-supplementary.pdf]

Omega-3 Questionnaire

Q1. Name (text entry)

____

Pre-questionnaire brief

When completing this questionnaire please be aware that we are considering a full spectrum of fish and seafood including and not limited to:

- Fresh
- Battered
- Frozen
- Breaded
- Tinned
- Fish cakes
- Fish burgers

Q2. Over the past 6 months, how many times have you eaten fish or shellfish in any form (including sushi prepared with fish or seafood) (Drop down list single answer)

- Never
- Less than 1 time each month
- 1 time each month
- 2-3 times each month
- 2 times each week
- 3-4 times each week
- 5-6 times each week
- 1 time each day
- 2 or more times each day

Q3. For what reasons have you consumed fish or shellfish in the last 6 months less than 2 times a week? (Multiple answers can be selected)

(Display this question if fish is consumed <2 times per week in Q2)

- Sensory barriers (taste, smell etc.)
- Environmental Impact
- Impact on body composition
- Impact on performance
- Allergy
- Risk of contaminants
- Not aware of possible benefits
- Health and nutritional beliefs
- Cost
- Cooking skills

- Accessibility
- Other (please state) ________________

Q4. Each time you ate fish or shellfish, how much did you eat? (Drop down list single answer)

(Display this question if 'Never' is not selected in Q2)

- Less than a fillet or less than 4 pieces of sushi
- About 1 fillet or 4-14 pieces of sushi
- More than 1 fillet or more than 14 pieces of sushi

Q5. Please check off the types of fish or shellfish you eat most frequently (Check off as many as are appropriate for you). (multiple answers can be selected)

(Display this question if 'Never' is not selected in Q2)

- Cod
- Crab
- Hake
- Haddock
- Herring
- Lobster
- Mackerel
- Mussels
- Oysters
- Salmon
- Scallops
- Trout
- Tuna
- Other (please specify) ________________

Q6. Over the past 6 months, how many times have you eaten walnuts? (Drop down list single answer)

(skip to Q8 if 'Never' is selected)

- Never
- Less than 1 time each month
- 1 time each month
- 2-3 times each month
- 2 times each week
- 3-4 times each week
- 5-6 times each week
- 1 time each day
- 2 or more times each day

Q7. Each time you ate walnuts, how much did you eat? (Drop down list single answer)

- Less than 10 nut halves (<30 grams)
- 10-20 nut halves (30-60 grams)
- More than 20 nut halves (>60 grams)

Q8. Over the past 6 months, how many times have you used rapeseed oil? (Drop down list single answer)

(skip to Q10 if 'Never' is selected)

- Never
- Less than 1 time each month
- 1 time each month
- 2-3 times each month
- 2 times each week
- 3-4 times each week
- 5-6 times each week
- 1 time each day
- 2 or more times each day

Q9. Each time you used rapeseed oil, how much did you use? (Drop down list single answer)

- Less than 1 teaspoon
- 1-2 teaspoons
- 1 tablespoon
- 1-2 tablespoons
- More than 2 tablespoons

Q10. Over the past 6 months, how many times have you eaten flaxseed? (Drop down list single answer)

(skip to Q12 if 'Never' is selected)

- Never
- Less than 1 time each month
- 1 time each month
- 2-3 times each month
- 2 times each week
- 3-4 times each week
- 5-6 times each week
- 1 time each day
- 2 or more times each day

Q11. Each time you ate flaxseed, how much did you eat? (Drop down list single answer)

- Less than 1 teaspoon
- 1-2 teaspoons
- 1 tablespoon
- 1-2 tablespoons
- More than 2 tablespoons

Q12. Over the past 6 months, how many times have you used flaxseed oil? (Drop down list single answer)

(skip to Q14 if 'Never' is selected)

- Never
- Less than 1 time each month
- 1 time each month
- 2-3 times each month
- 2 times each week
- 3-4 times each week
- 5-6 times each week
- 1 time each day
- 2 or more times each day

Q13. Each time you used flaxseed oil, how much did you use?

- Less than 1 teaspoon
- 1-2 teaspoons
- 1 tablespoon
- 1-2 tablespoons
- More than 2 tablespoons

Q14. Over the past 6 months, how many times have you eaten chia seeds? (Drop down list single answer)

(skip to Q16 if 'Never' is selected)

- Never
- Less than 1 time each month
- 1 time each month
- 2-3 times each month
- 2 times each week
- 3-4 times each week
- 5-6 times each week
- 1 time each day
- 2 or more times each day

Q15. Each time you used chia seeds, how much did you use?

- Less than 1 teaspoon
- 1-2 teaspoons
- 1 tablespoon
- 1-2 tablespoons
- More than 2 tablespoons

Q16. Over the past 6 months, how many times have you used vegetable margarine? (Drop down list single answer)

(skip to Q19 if 'Never' is selected)

- Never
- Less than 1 time each month
- 1 time each month
- 2-3 times each month
- 2 times each week
- 3-4 times each week
- 5-6 times each week
- 1 time each day
- 2 or more times each day

Q17. Each time you used vegetable margarine, how much did you use? (Drop down list single answer)

- Less than 1 teaspoon
- 1-2 teaspoons
- 1 tablespoon
- 1-2 tablespoons
- More than 2 tablespoons

Q18. Which margarine (brand and product) did you use? (text entry)

____

Q19. Where have you sourced your current information on omega-3? (Drop down list multiple answer)

- Internet
- Qualified nutritionist/ dietician
- Other members of staff
- Another athlete
- Family/ friend
- Other (please specify) ________________

Q20. Do you currently supplement, or have you supplemented over the last 6 months with any omega-3 containing product? (Multiple choice)

(if 'no' is selected skip to Q23)

- Yes
- No

Q21. Do you currently supplement, or have you supplemented over the last 6 months with any omega-3 containing product? (Multiple choice)

- 1
- 2
- 3
- 4
- 5

Q22. If you currently take any omega-3 supplements, please provide some details in the table below. If you do not know some of the information, please fill out what you can.

Examples include: omega-3, fish oil, algal oil and cod liver oil.

(Display if answered ‘Yes’ to Q20)

(number of choices displayed is reflected by answer provided in Q23)

	Supplement Name	Brand name	Frequency of use	Dose	Dose unit	Main reasons for use
	e.g. fish oil	E.g. Optimum Nutrition		e.g. 1,000 or N/A if dose unknown		
Supplement 1	Free text	Free text	Drop down list single answer: options:<br>▫ Less than 1 time each month<br>▫ 1 time each month<br>▫ 2-3 times each month<br>▫ 1 time each week<br>▫ 2 times each week<br>▫ 3-4 times each week<br>▫ 5-6 times each week<br>▫ 1 time daily<br>▫ 2 or more times daily	Free text	Drop down list single answer:<br>▫ Mg<br>▫ g<br>▫ Ml<br>▫ N/A	Drop down list multiple answer: Options:<br>▫ Enhanced recovery<br>▫ Cardiovascular health<br>▫ Brain function<br>▫ Support fat loss<br>▫ Reduce inflammation<br>▫ General Health<br>▫ Prefer not to say<br>▫ Don’t know

Q23. Have you consumed an omega-3 enriched products in the past 6 months that was not mentioned in the questionnaire? (e.g. eggs, milk, Enhanced Recovery Omega-3 Sports Drink) (Multiple choice)

If ‘no’ the questionnaire ends

Yes

No

Q24. Which product did you consume and how often did you consume it? (text entry)

____

If you have any comments about the questionnaire in general or about your diet in the past six months, you can leave further information below:

____
____
____
____
____

Thank you for completing this survey. Your responses have been recorded.

Thank you for your time and participation
